# Primary Mesenteric Well-Differentiated Inflammatory Liposarcoma With Mucosal Extension: A Lesion With High Risk for Misdiagnosis

**DOI:** 10.1155/cris/9162938

**Published:** 2025-04-19

**Authors:** William Abel, Christopher J. Peterson, David P. Lebel, Douglas J. Grider

**Affiliations:** ^1^Department of Internal Medicine, Virginia Tech Carilion School of Medicine, Roanoke, Virginia, USA; ^2^Gastroenterology and Hepatology Division, Department of Internal Medicine, Virginia Tech Carilion School of Medicine, Roanoke, Virginia, USA; ^3^Department of Basic Science Education, Virginia Tech Carilion School of Medicine, Roanoke, Virginia, USA; ^4^Dominion Pathology Associates, Roanoke, Virginia, USA

**Keywords:** lipoblasts, MDM2, mesenteric liposarcoma, mesenteric mass, plasma cells, well-differentiated liposarcoma

## Abstract

Well-differentiated liposarcomas are common retroperitoneal lesions, but exceedingly rare when primary to the small bowel mesentery, with only a handful of cases reported in the literature. Presented is a patient with a primary mesenteric well-differentiated inflammatory liposarcoma with mucosal extension at high risk for misdiagnosis. A broad differential diagnosis with careful histopathologic observation, ancillary immunohistochemical studies, and fluorescent in situ hybridization for MDM2 amplification are key to make a correct diagnosis. This is especially true if such a lesion was first noted in the lamina propria on histopathology from an endoscopic mucosal biopsy.

## 1. Introduction

Primary mesenteric liposarcoma is a rare malignancy, with only a handful of cases reported in the literature. Liposarcoma originates from adipose tissue and can rarely involve the gastrointestinal tract and mesentery [1]. The World Health Organization lists five liposarcoma classifications: well-differentiated, de-differentiated, myxoid, pleomorphic, and myxoid pleomorphic [2]. Well-differentiated liposarcoma is further divided into four subtypes: adipocytic, sclerosing, inflammatory, and spindle cell [2–5]. Although no randomized trials have been performed given the rarity of well differentiated inflammatory liposarcoma, disease-free survival ranges from 42.3% to 87.1% depending on the subtype, highlighting the importance of proper diagnosis for prognostication and treatment [6]. Presented is a case of well-differentiated inflammatory liposarcoma with an excellent treatment outcome.

## 2. Case Report

**Figure 1:**
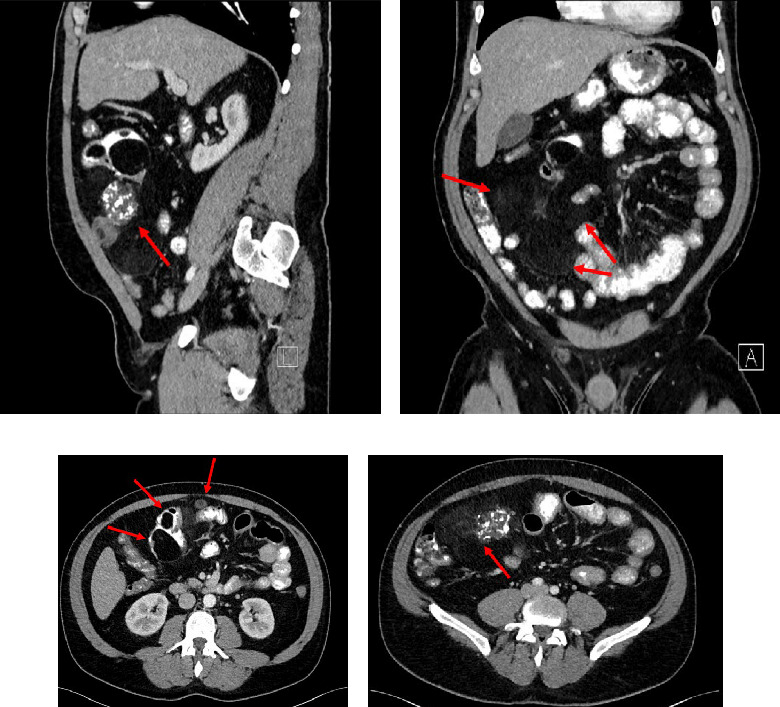
(a–d) CT scan abdomen, mass with both fatty density, and bony components denoted by arrows.

**Figure 2:**
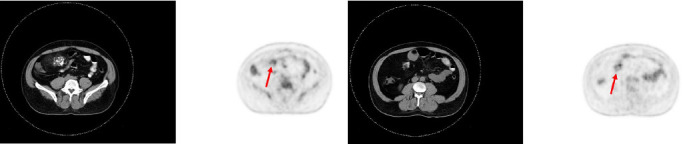
(a, b) CT scan of the abdomen juxtaposed with corresponding PET scan.

A 47-year-old male with a past medical history notable for hypertension and gout presented to his primary care physician for an episode of band-like mid-abdominal pain with radiation to the back, fever, and diarrhea. He was treated initially with conservative measures including proton pump inhibitors with minimal relief. Initial labs were significant for a white blood cell (WBC) count of 12.7 K/uL, a hemoglobin of 13.2 g/dL, and a C-reactive protein (CRP) of 41. A subsequent computed tomography (CT) scan revealed an approximately 12 cm (in its largest dimension) ill-defined lipomatous mesenteric mass with a 4.3 cm × 4.2 cm solid nodular component with calcifications in the right lower abdomen suspicious for liposarcoma, but with no proximal dilation to indicate obstruction (Figure 1). He was subsequently referred to medical and surgical oncology for further evaluation. PET/CT scan noted minimal FDG avidity within the right lower quadrant mass and no other metabolically active sites (Figure 2). Further interview revealed a 40-pound weight loss over 6 months. There was no history of malignancies, although family history was significant for lymphoma in the patient's mother. Exploratory laparotomy with possible small bowel resection and primary anastomosis was planned by surgical oncology based on the lack of metastatic disease and no small bowel dilation. Intraoperatively, the 12 cm pedunculated lipomatous mass emanating from the jejunum was identified approximately 78 cm from the ligament of Treitz. Approximately 5 cm of small bowel proximal and distal to the mass was resected for a total of 10 cm of small bowel resection in addition to resection of the mass itself. A 12.4 cm × 10.3 cm × 7.1 cm complex, predominantly fatty mass, with a 4.3 cm × 4.2 cm solid nodular component with calcifications was removed. Intraoperative frozen section of the small bowel margins was free of disease, and thus, primary anastomosis of the small bowel was performed. Postoperatively, the patient recovered without surgical complications and was discharged on postoperative day 2.

**Figure 3:**
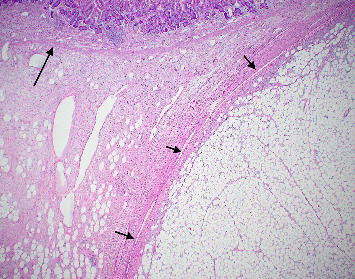
Resection specimen histopathology with a well-differentiated lipocytic lesion noted (short arrows) juxtaposed to a spindle cell proliferation with intervening lipocytic cells extending into the mucosal lamina propria (long arrow shows extension through the muscularis mucosa into the lamina propria; H&E 10x).

**Figure 4:**
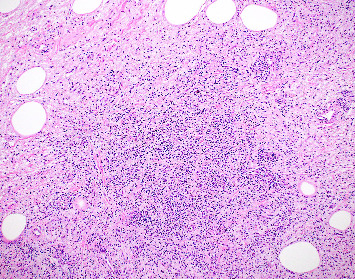
Nodular lymphoplasmacytic infiltrate amongst the spindle cells with associated lipocytes (H&E 10x).

**Figure 5:**
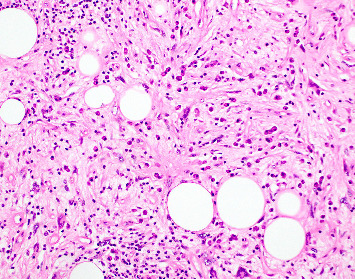
Plasma cell rich area amongst the spindle cells with associated lipocytes (H&E 20x).

**Figure 6:**
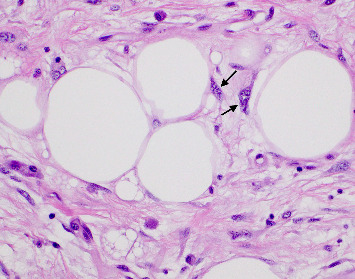
Lipoblasts denoted at arrows (H&E 60x).

**Figure 7:**
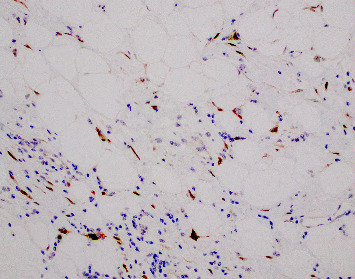
MDM2 immunohistochemical overexpression demonstrated by nuclear positivity (40x).

The resected specimen showed a mesenteric mass extending through the muscularis propria into the submucosa with focal extension into the lamina propria (Figure 3). On histopathology, the mass showed defined areas of well-differentiated lipocytes separated from other juxtaposed areas showing a spindled cell component with variable and somewhat nodular lymphoplasmacytic infiltrate and easily identified lipoblasts (Figures 3–6). Focal storiform fibrosis but no obliterative phlebitis was noted, including on an elastic stained tissue section, excluding IgG4 disease. However, some of the plasma cells were IgG4 positive. In distinct areas, the mass was associated with metaplastic bone formation intertwined with atypical-appearing lipoblasts. Both the lipocytes and lipoblasts were S-100 protein and MDM2 positive (Figure 7). In addition, fluorescence in situ hybridization for MDM2 showed amplification. These ancillary studies confirmed a diagnosis of well-differentiated liposarcoma, inflammatory phenotype. Resected lymph nodes (14 in total) were all negative for liposarcoma. The neoplasm was staged as T2a N0 grade 1 well-differentiated liposarcoma with inflammatory subclassification.

After shared decision-making, the patient opted against chemotherapy, which was determined to have minimal benefit in this case. He received surveillance CT imaging at 2 months and then 6 months, with the interval extended to and maintained at a 12-month interval for the foreseeable future. Surveillance imaging was negative for tumor recurrence and no palpable mass was present on follow-up abdominal examination.

## 3. Discussion

Primary inflammatory mesenteric liposarcoma is a highly rare form of malignancy with only around 30 cases documented in the literature [7]. This patient's symptomatic presentation was typical of patients, with weight loss and abdominal pain as common presenting symptoms as was his diagnosis during the fifth decade of life [4, 5]. In addition to the rarity of this pathology, this case highlights the diagnostic dilemma that might be confounding should a sample of such a sarcoma be first evaluated from an endoscopic sample.

The initial gross pathologic examination revealed a large, predominately fatty-appearing tumor, with some nodules and calcifications, arising from the small bowel mesentery with extension into the bowel wall. Histopathology corroborated this, showing an adipocyte-rich, spindled cell proliferation with scattered lipoblasts and areas of storiform fibrosis without obliterative phlebitis. Interestingly, in the section of the tumor that was adherent and extending into the bowel wall, there was invasion beyond the muscularis propria reaching as far as the lamina propria. The histopathologic differential diagnosis includes dedifferentiated liposarcoma, IgG4-related disease, inflammatory myofibroblastic tumor, nodular fasciitis, and pleomorphic lipoma/spindle cell lipoma [3, 8, 9].

Given that inflammatory liposarcoma is such a rare form of well-differentiated liposarcoma (2% of cases), is typically retroperitoneal, and unlikely to invade the bowel wall, it is a highly unexpected diagnosis for this presentation. Thus, clinical suspicion along with histopathologic acumen to select the best ancillary studies to arrive at the correct diagnosis is necessary [3]. The adipocyte and spindle cell rich appearance of the tissue placed pleomorphic lipoma/spindle cell lipoma as well as differentiated liposarcoma within the differential diagnosis [9]. Lipoblasts may be found in atypical spindle cell lipomatous tumors (generally not found in lipomas) placing them within the differential as well [9, 10]. IgG4-related disease or inflammatory myofibroblastic tumor typically do not have lipoblasts and are not MDM2 positive. Further, obliterative phlebitis was not found, even on an elastic tissue stain, and the spindled cells did not have a distinct myofibrolastic histopathologic appearance of immunohistochemical profile, excluding both IgG4 disease and myofibroblastic tumor, respectively [8].

MDM2 by both immunohistochemical stain and FISH were very useful in further classifying this as liposarcoma, being overexpressed by both methods [11]. The junction between the adipocytes and spindle cells within a de-differentiated liposarcoma is particularly revealing of its de-differentiated nature with many bizarre cells. This histopathologic finding was not noted within the surgical specimen obtained from our patient, with the morphology being consistent with the well differentiated form [10]. Thus, the diagnosis of inflammatory liposarcoma arising from the mesentery was reached.

The advantages of having a resection specimen were apparent in this case as a large amount of tissue showing bowel wall and tumor borders was needed to make the correct diagnosis. A foreseeable dilemma, however, is in the rise of EUS-guided biopsy, since the mass was both abutting and invading into the luminal side of the small bowel. While EUS-guided biopsy likely would have been possible given invasion beyond the muscularis and into the lamina propria, the specimen may not have given the amount of tissue and the variety of sampling needed to make the diagnosis. Thus, a full surgical specimen is very often vital in making the final diagnosis.

## Data Availability

The data for this case report is available by contacting Dominion Pathology Associates under the address for the corresponding author above.

## References

[1] Kindblom L. G., Meis-Kindblom J. M., Enzinger F. M. (1995). Variants of Liposarcoma. *The American Journal of Surgical Pathology*.

[2] Sbaraglia M., Bellan E., Dei Tos A. P. (2021). The 2020 WHO Classification of Soft Tissue Tumours: News and Perspectives. *Pathologica*.

[3] Kraus M. D., Guillou L., Fletcher C. D. M. (1997). Well-Differentiated Inflammatory Liposarcoma: An Uncommon and Easily Overlooked Variant of a Common Sarcoma. *The American Journal of Surgical Pathology*.

[4] Choi J. H., Ro J. Y. (2020). Retroperitoneal Sarcomas: An Update on the Diagnostic Pathology Approach. *Diagnostics (Basel)*.

[5] Wang G. Y., Lucas D. R. (2018). Dedifferentiated Liposarcoma With Myofibroblastic Differentiation. *Archives of Pathology & Laboratory Medicine*.

[6] Edagawa M., Haratake N., Shimamatsu S. (2017). Surgical Resection of a Well-Differentiated Inflammatory Liposarcoma of the Middle Mediastinum: A Case Report. *Journal of Thoracic Disease*.

[7] Garg P. K., Jain B. K., Dahiya D., Bhatt S., Arora V. K. (2014). Mesenteric Liposarcoma: Report of Two Cases with Review of Literature. *Journal of Gastrointestinal Cancer*.

[8] Taylor M. S., Chougule A., MacLeay A. R. (2019). Morphologic Overlap Between Inflammatory Myofibroblastic Tumor and IgG4-Related Disease: Lessons From Next-generation Sequencing. *American Journal of Surgical Pathology*.

[9] Goldblum J. R., Folpe A. L., Weiss S. W., Enzinger F. M. (2014). *Enzinger and Weiss’s soft tissue tumors*.

[10] Thway K. (2019). Well-Differentiated Liposarcoma and Dedifferentiated Liposarcoma: An Updated Review. *Seminars in Diagnostic Pathology*.

[11] Kammerer-Jacquet S. F., Thierry S., Cabillic F. (2017). Differential Diagnosis of Atypical Lipomatous Tumor/Well-Differentiated Liposarcoma and Dedifferentiated Liposarcoma: Utility of p16 in Combination With MDM2 and CDK4 Immunohistochemistry. *Human Pathology*.

